# Kinetics of IgA Subtypes and Cytokines in Respiratory Secretions Following Immunization With COVID‐19 Mucosal Vaccine

**DOI:** 10.1002/jmv.70638

**Published:** 2025-10-13

**Authors:** Weixin Chen, Juan Li, Shuang Bai, Ao Zhang, Qun Zheng, Junnan Zhang, Wenwen Lan, Yihan Zhang, Wei Yao, Wei Zhao, Jiang Wu

**Affiliations:** ^1^ Beijing Key Laboratory of Surveillance, Early Warning and Pathogen Research on Emerging Infectious Diseases Beijing Research Center for Respiratory Infectious Diseases, Beijing Center for Disease Prevention and Control Beijing China; ^2^ Experimental Center for Basic Medical Teaching, School of Basic Medical Sciences Capital Medical University Beijing China

**Keywords:** COVID‐19 mucosal vaccine, cytokine profiling IgA subclass kinetics, respiratory mucosal immunity

## Abstract

The COVID‐19 pandemic highlights the need for mucosal vaccines inducing respiratory secretory IgA (sIgA), as traditional intramuscular vaccines primarily elicit systemic IgG with limited mucosal protection. This study systematically compared mucosal immune responses induced by two authorized COVID‐19 mucosal vaccines: an orally aerosolized adenovirus vector‐based vaccine (Ad5‐nCoV) and an intranasal live‐attenuated influenza virus vector‐based vaccine (dNS1‐RBD). We longitudinally assessed IgA, IgA1, and IgA2 antibody titers against SARS‐CoV‐2 RBD/Spike protein, alongside cytokine profiles, in nasal secretions and sputum from 40 participants at 7, 14, 28 days, and 3/6 months post‐immunization. The orally aerosolized vaccine exhibited superior mucosal immunogenicity, with peak IgA positive conversion rate of 50% (nasal) and 65% (sputum) vs. 30% (nasal) and 40% (sputum) for the intranasal vaccine. Notably, the orally aerosolized vaccine induced sustained IgA2 dominance (> 50%) in sputum at 6 months, whereas the intranasal vaccine showed transient IgA1 predominance followed by decline. Despite these differences, both vaccines elicited modest overall mucosal responses, with only IL‐6 showing significant intergroup variation (*p* < 0.01). This is a study to demonstrate compartmentalized IgA subclass dynamics between upper (nasal) and lower (sputum) respiratory tracts following mucosal vaccination. Our findings highlight the need for optimized mucosal vaccine formulations to enhance respiratory immunity, providing critical insights for developing next‐generation COVID‐19 vaccines targeting the complex mucosal immune microenvironment.

## 1 Introduction

Coronavirus disease 2019 (COVID‐19), caused by severe acute respiratory syndrome coronavirus 2 (SARS‐CoV‐2), remains a global public health threat despite the widespread deployment of vaccines [1]. While intramuscular administered vaccines effectively induce systemic IgG response and reduce disease severity [2, 3, 4]. They fail to establish mucosal immunity in the respiratory—the primary site of viral entry [5]. This critical limitations has driven the development of mucosal vaccines, which aim to elicit secretory IgA (sIgA) antibodies at respiratory mucosal surfaces [6].

Two mucosal COVID‐19 vaccines have been authorized in China: an intranasal live‐attenuated influenza virus vector‐based vaccine (dNS1‐RBD) encoding the receptor binding domain (RBD) [7]. An orally aerosolized adenovirus vector‐based vaccine(Ad5‐nCoV), expressing full‐length SARS‐CoV‐2 spike protein [8]. Despite promising preclinical data, the mucosal immune responses induced by these vaccines remain incompletely characterized, particularly regarding the dynamics of IgA subclasses and cytokine profiles across respiratory compartments [9].

Mucosal immunity is orchestrated by a complex network of humoral and cellular responses, with sIgA serving as the primary effector molecule [10, 11, 12]. Humans possess two IgA subclasses with distinct biological properties: IgA1, characterized by an extended hinge region facilitating antigen binding, and IgA2 which exhibits enhanced resistance to bacterial proteases [13]. While IgA1 predominates in systemic circulation (> 90%), the IgA1/IgA2 ratio varies across mucosal sites. IgA2 is enrich in the distal gastrointestinal tract and saliva glands, whereas IgA1 dominates in nasal mucosal [14, 15]. This compartmentalization suggests differential roles in respiratory defense, yet their kinetics following mucosal vaccination remain unelucidated.

Cytokines play a pivotal role in regulating mucosal B cell differentiation and IgA class switching. Th1 cytokines (e.g., IFN‐γ) promote cellular immunity, while Th2/Th17 cytokines (e.g., IL‐4, IL‐17) enhance sIgA secretion [16, 17]. Importantly, viral vectors through TLR9, including Th1‐skewed responses, whereas influenza virus activated TLR7/8, promoting Th2/Th17 polarization [18, 19]. These divergent cytokine profiles may differentially regulate IgA subclass distribution and tissue‐specific secretion, yet this hypothesis remains untested.

In this study, we comprehensively compared the mucosal immunogenicity of the two authorized COVID‐19 mucosal vaccines by analyzing IgA, IgA1, and IgA2 responses against SARS‐CoV‐2 RBD/Spike protein, alongside 15 cytokines, in nasal secretions and sputum from 40 participants at 7, 14, 28 days, and 3/6 months post‐immunization. This is the first study to systematically evaluate vector‐specific mucosal immune compartmentalization, providing critical insights for optimizing respiratory mucosal vaccines.

## 2 Methods

### 2.1 Study Design and Participants

Participants recruited in this study must meet the following eligibility criteria: aged between 18−65 years, in good general health, having uniformly received three doses of inactivated vaccine; having a history of only one prior SARS‐CoV‐2 infection with more than 6 months since their last infection at the time of enrollment, and having no history of severe allergic reactions or significant comorbidities that may affect vaccine‐induced immune responses.

To ensure the validity of the study, participants were excluded if they had received any other COVID‐19 vaccine within the preceding 3 months, had a confirmed or suspected COVID‐19 infection at the time of enrollment, or had a compromised immune system due to conditions such as HIV/AIDS or immunosuppressive therapy. Additionally, pregnant or breastfeeding women were excluded to mitigate potential risks to the fetus or infant.

**Figure 1:**
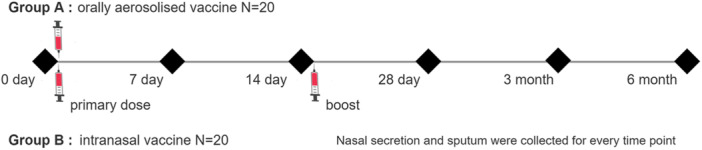
Study design and the sample collection.

All the participants were randomly allocated to two groups for this study. Group A received a single dose of the Adenovirus Vector Vaccine (Ad5‐nCoV) developed by CanSino (Tianjin, China). The replication‐deficient human adenovirus type 5 (Ad5) vector was engineered to express the full‐length SARS‐CoV‐2 spike protein (GenBank: MN908947.3) under the CMV promoter. The E1 and E3 regions were deleted to prevent viral replication in human cells [20, 21]. Each 0.1 mL dose was aerosolized over 25 s. Participants inhaled the aerosol in a single breath, followed by a 5‐second breath hold [22] Group B received two doses of the intranasal vaccine produced by Wantai (Beijing, China), with a 14‐day interval. The vaccine was constructed using a double‐attenuated influenza virus vector (CA4‐dNS1) with deletion of the NS1 gene to enhance safety. The receptor‐binding domain (RBD) of SARS‐CoV‐2 spike protein (GenBank: MN908947.3) was inserted into the NS gene segment of the influenza virus A/California/07/2009 (H1N1) backbone using reverse genetics [7, 23]. A 0.2 mL dose (bilateral nasal spray, 0.1 mL per nostril) was administered using a single‐use nasal applicator (Wantai Medical) [24]. Nasal secretion and sputum samples were systematically collected from all participants at six specific time points to assess variations in IgA, IgA1, and IgA2 levels. These time points included the pre‐immunization phase (baseline), followed by intervals at days 7, 14, and 28, and 3‐ and 6‐months postvaccination (Figure 1). Sample collection was conducted under standardized conditions to ensure data reliability and comparability.

Nasal secretion samples were also analyzed for the presence of SARS‐CoV‐2 ribonucleic acids at all time points.

The study protocols were approved by the Ethics Committee of the Beijing Center for Disease Control and Prevention (Ethics No. 14th, 2022), and written informed consent was obtained from all the participants before their involvement in the study.

### 2.2 Nasal Secretion Collection

Nasal secretion collection followed the methodology outlined by Watelet et al. [25]. Medical‐grade polyvinyl alcohol (PVA) sponges (Kuaijie, Beijing, China) served as the collection apparatus. These sponges were precisely cut into strips approximately 2.5 cm in length and 0.5 cm in width using sterile scissors. Two strips were placed into a sterile 15 mL centrifuge tube, with one strip designated for each nasal cavity. The pre‐collection weight of the strips was determined by marking the tubes accordingly.

Each PVA sponge strip was gently inserted into the base of the nasal cavity. After an absorption period of 5–10 min, the fully saturated sponges were carefully extracted using forceps and placed into a 15 mL centrifuge tube, which was then sealed to prevent evaporation. The post‐collection weights of the centrifuge tubes were recorded.

Next, 2 mL 0.9% saline solution was added to the tube containing the expanded sponges. The tubes were inverted and maintained at 4°C for 2 h to allow the sponges to completely absorb saline. Afterward, the sponges were transferred into a 5 mL syringe. The syringe plunger was depressed to compress the sponges, and a segment of the plunger was removed to facilitate centrifugation. The samples were then centrifuged at 1500*g* for 10 min to ensure complete recovery of the liquid. Following centrifugation, the syringe components were disassembled, and the collected samples were aliquoted and stored at −40°C for subsequent analysis.

### 2.3 Sputum Samples Collection

Sputum samples were collected according to the methodology outlined by Guardline [26]. The participants were instructed to rinse their mouths with water and clear their nasal passages to remove debris. They then inhaled hypertonic saline (3.0% or 4.5%) through nebulization for 10 min. Subsequently, the participants rinsed their mouths and cleared their nasal passages again before being instructed to cough vigorously to expel sputum into a collection tube. If the initial attempt did not yield an adequate sputum sample, the nebulization process was repeated for another 10 min, as needed, until a sufficient sample was obtained. The total nebulization duration did not exceed 30 min. The collected sputum specimens were treated with sputum digestion fluid to prepare them for further analysis.

### 2.4 RNA Extraction and Determination of SARS‐CoV‐2 Virus Infection by RT‐PCR

All nasal secretion samples were subjected to ribonucleic acid testing to detect SARS‐CoV‐2. Ribonucleic acids were extracted using the extraction or purification reagents provided by Changchun Bokun Biological Technology Co. Ltd. This extraction process was conducted using an automated ribonucleic acid extraction instrument (Thermo Scientific) to ensure consistency and efficiency. Following extraction, SARS‐CoV‐2 was detected using a 2019‐nCoV ribonucleic acid detection kit (fluorescent PCR method) developed by Daan Gene Co. Ltd.

### 2.5 Meso Scale Discovery Assays to Detect—IgA, IgA1, and IgA2 Levels and Cytokines in Nasal Secretion and Sputum

The V‐PLEX SARS‐CoV‐2 Panel 2 (IgA) Kit (Meso Scale Diagnostics LLC; Rockville, MD, USA, K15385U‐2) was used to quantify IgA antibodies targeting the SARS‐CoV‐2 S1 RBD, Spike, and N proteins. The assays were performed according to the manufacturer's instructions. Briefly, plates were prepared and incubated with diluted nasal secretions or sputum samples, followed by incubation with an electrochemiluminescent label (SULFO‐TAG anti‐human IgA antibody). The analysis was conducted using MESO QuickPlex SQ 120MM (Meso Scale Diagnostics LLC; Rockville, MD, USA). Recombinant protein standards were serially diluted (1:4) in sample diluent, with 3 technical replicates per dilution. Calibration curves were generated using a 4‐Parameter Logistic (4‐PL) regression model (*R*² > 0.99) to interpolate sample concentrations. This assay was used for relative quantification of analyte levels between study groups and time points, rather than qualitative determination of positivity/negativity. Titers were determined with reference to the National Standard for SARS‐CoV‐2 Neutralizing Antibodies using human plasma (catalog number 280034‐202102, National Institutes for Food and Drug Control, China) to create a calibration curve. The results are expressed in arbitrary units per milliliter (AU/mL). The use of arbitrary units allows for semi‐quantitative comparison of immune response magnitudes across different time points and between study groups, without defining strict positivity thresholds.

Detection of the IgA1 and IgA2 subclasses followed a similar protocol, with key variations in the choice of secondary antibodies specific to each subclass. For IgA1 antibodies, we used a product from Southern Biotech (Catalog No. 9130‐01), whereas IgA2 antibodies were obtained from Abcam (Catalog No. ab309578). The same relative quantification approach was applied to IgA subclass analysis, with standard curves generated for each subclass‐specific assay to ensure accurate comparison of subclass distribution patterns.

The V‐PLEX Custom Human Biomarkers Kit (Meso Scale Diagnostics LLC, Rockville, MD, USA, K151A9H‐2) was used to detect a range of cytokines, including Interleukin‐2 (IL‐2), Interleukin‐4 (IL‐4), Interleukin‐6 (IL‐6), Interleukin‐10 (IL‐10), Interleukin‐12 p70 (IL‐12p70), Interleukin‐13 (IL‐13), Interleukin‐17 (IL‐17A), Interleukin‐21 (IL‐21), Interleukin‐22 (IL‐22), Interleukin‐23 (IL‐23), and Interferon‐gamma (IFN‐γ). Additionally, the U‐PLEX transforming growth factor‐beta (TGF‐β) Combo (hu) SECTOR Kit (Meso Scale Diagnostics LLC; Rockville, MD, USA, K15241K‐2) was used to detect Transforming Growth Factor‐beta 1,2,3 (TGF‐β1, TGF‐β2, and TGF‐β3). U‐PLEX Custom Immuno‐Oncology Group 1 (hu) Assays (Meso Scale Diagnostics LLC; Rockville, MD, USA, K151AEM‐2) were used to identify Interleukin‐5 (IL‐5), Interleukin‐18 (IL‐18), and Interferon alpha‐2a (IFN‐α2a). Cytokine concentrations were determined using cytokine‐specific standard curves prepared from recombinant protein standards (provided in each kit), following the same 4‐PL regression model for relative 1/2 quantification. All cytokine assays were performed with 2 technical replicates, and results below the limit of detection (LOD) were reported as LOD/2 for statistical analysis.

### 2.6 Positive Conversion Rate Calculation

The antibody titers detected on Day 0 (pre‐vaccination) were defined as the baseline.

Positive conversion was operationally defined as a postvaccination titer at least double the pre‐vaccination baseline titer. For each sample, if the postvaccination titer met or exceeded this twofold increase, it was classified as “converted.” The positive conversion percentage at each time point was then calculated as the proportion of converted samples within the total number of samples tested for that group.

### 2.7 Statistical Analyses

*χ* ^2^ tests were used to assess significant differences in the seroconversion rates among the different vaccines. Descriptive statistical analysis was conducted to summarize the geometric mean antibody titers and seropositivity rates for each vaccine. *T*‐test was used to compare the IgA, IgA1 and IgA2 titers at different time points. Using GraphPad Prism version 8.0.2 for Windows, GraphPad software, Boston, MA, www.graphpad.com, and a *p*‐value of less than 0.05 was considered a statistically significant difference.

## 3 Results

### 3.1 Participant Demographics

To ensure valid comparisons of immune response between the two vaccine groups, demographic data were collected to assess potential confounding factors (e.g., age, gender) that might influence vaccine efficacy. Baseline SARS‐CoV‐2 ribonucleic acid (RNA) testing was performed to exclude participants with pre‐existing infections. A total of 40 eligible participants were enrolled, with 20 assigned to the orally aerosolized vaccine group (Group A) and 20 to the intranasal vaccine group (Group B).

SARS‐CoV‐2 RNA test were negative for all participants at all time points. Participant demographics are summarized in Table 1. Group A included 8 males and 12 females with a mean age of 51.15 years, while Group B comprised 10 males and 10 females with a mean age of 41 years. Statistical analysis revealed no significant difference in gender distribution between the two groups (*p* = 0.7512); however, a significant difference in age was observed (*p* = 0.0118).

**Table 1:** Demographic characteristics of participants.

Characteristic	Orally	Intranasal	Total	*p*
Overall, *n* (%)	20 (50)	20 (50)	40 (100)	
Average age	51.15	41	46.1	0.0118
Sex, *n* (%)				
Male	8 (40)	10 (50)	18 (45)	0.7512
Female	12 (60)	10 (50)	22 (55)	

### 3.2 Anti‐SARS‐CoV‐2 Specific IgA, IgA1, and IgA2 in Nasal Secretions and Sputum Samples

To characterize the kinetics of local immune responses following different vaccination routes, this study measured IgA, IgA1, and IgA2 antibody titers against SARS‐CoV‐2 RBD and Spike protein in nasal secretions and sputum samples from two cohorts: participants receiving the orally aerosolized vaccine (Group A) and the intranasal vaccine (Group B). Positive conversion was defined as a titer at least double the pre‐vaccination level due to baseline variations.

### 3.3 SARS‐CoV‐2 RBD‐Specific Antibody Responses

**Figure 2:**
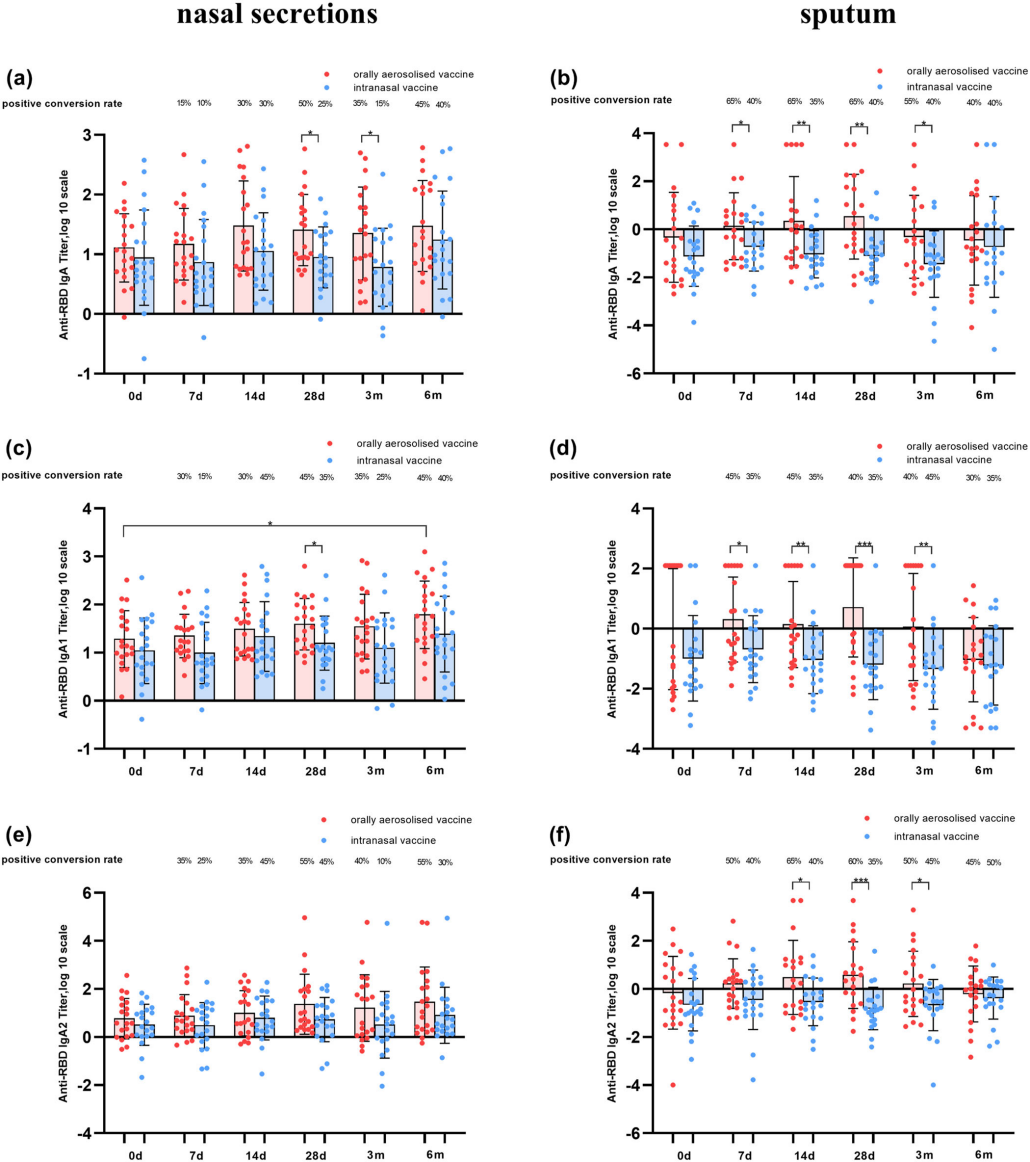
Antibody titers and positive conversion rates of IgA, IgA1 and IgA2 tested by SARS‐CoV‐2 RBD protein. (a, c, and e) represent the antibodies of IgA, IgA1, and IgA2 in nasal secretions, respectively. (b, d, and f) represent the antibodies of IgA, IgA1, and IgA2 in sputum, respectively. *p*‐values from *t*‐test, comparing each time point shown. *p < 0.05, **p < 0.01, ***p < 0.001.

For nasal secretions, total IgA of Group A exhibited an increasing positive conversion rate post‐immunization, peaking at 50% on Day 28. In contrast, Group B reached a 30% peak on Day 14 followed by a declined (Figure 2a). IgA1 of Group A achieved a 45% peak positive conversion rate at 28 days post‐immunization and maintained elevated levels, while Group B showed a similar Day 28 peak without sustained elevation (Figure 2c). IgA2 of Group A reached a 55% positive conversion rate on Day 28 with sustained elevation, whereas Group B peaked on Day 14 and subsequently declined. No statistically significant intergroup differences were observed at any time point (Figure 2e).

For sputum samples, despite lower absolute concentrations compared to nasal secretions, total IgA in sputum sample showed higher positive conversion rate. Group A reached 65% on Day 7 and stabilized, while Group B peaked at 40% on Day 7 and maintained levels between 35% and 40% (Figure 2b). IgA1 of both groups maintained positive conversion rates between 30% and 40% starting from Day 7 onwards, with Group A exhibiting numerically higher levels at multiple time points (Figure 2d). IgA2 of Group A sustained positive conversion rates between 50% and 65% from Day 7, while Group B maintained levels between 35% and 50%. Group A surpassed Group B at several time points (Figure 2f).

**Figure 3:**
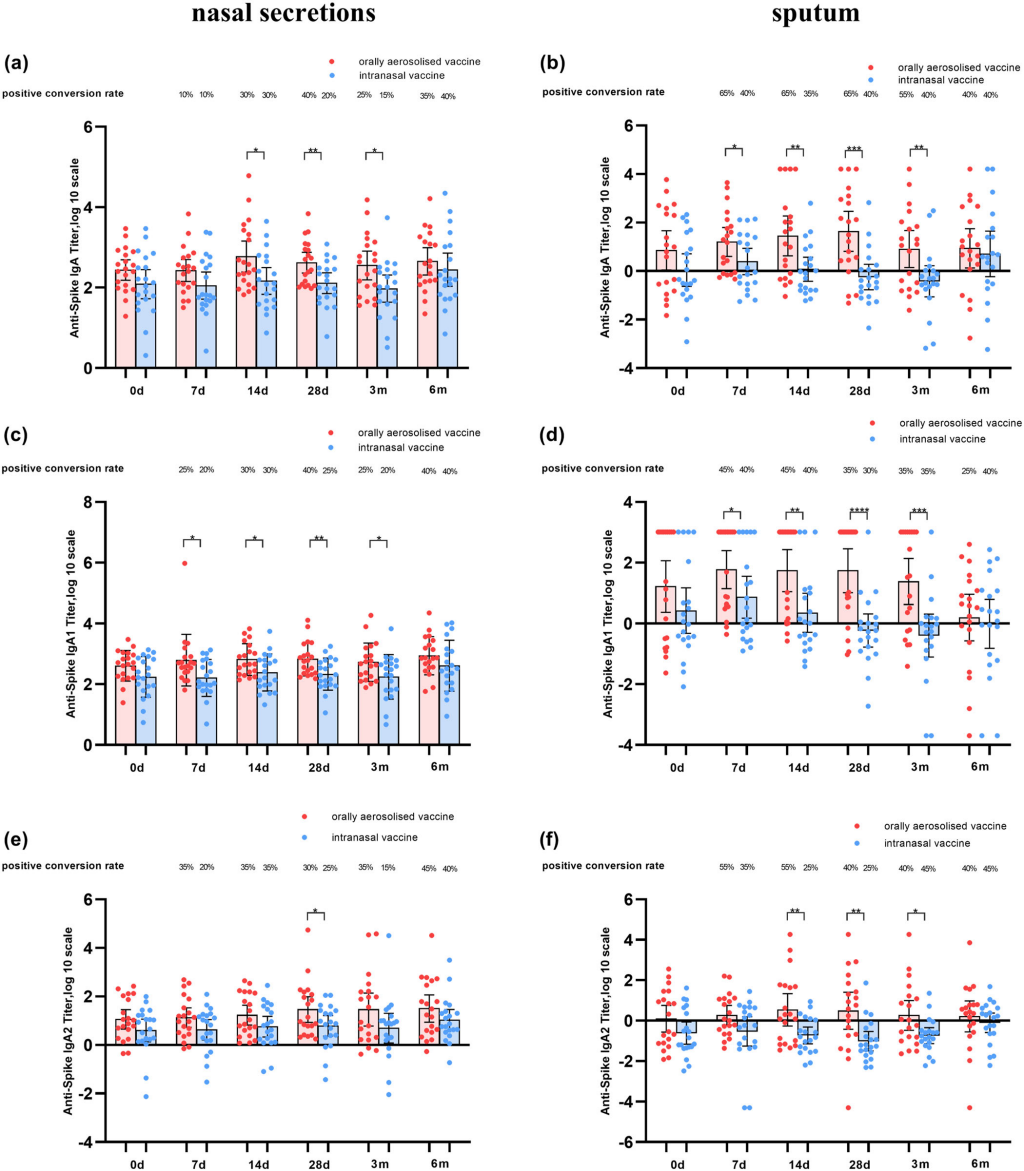
Antibody titers and positive conversion rates of IgA, IgA1 and IgA2 tested by SARS‐CoV‐2 Spike protein. (a, c, and e) represent the antibodies of IgA, IgA1, and IgA2 in nasal secretions, respectively. (b, d, and f) represent the antibodies of IgA, IgA1, and IgA2 in sputum, respectively. *p*‐values from *t*‐test, comparing each time point shown. *p < 0.05, **p < 0.01, ***p < 0.001.

Analogous tests on the spike protein results are shown in Figure 3.

### 3.4 Proportion of IgA1 and IgA2 in Nasal Secretions and Sputum

IgA1 and IgA2 exhibit distinct structural and functional properties, and their proportional balance in mucosal secretions provides critical insights into subtype‐specific immune responses and potential long‐term protective mechanisms.

**Figure 4:**
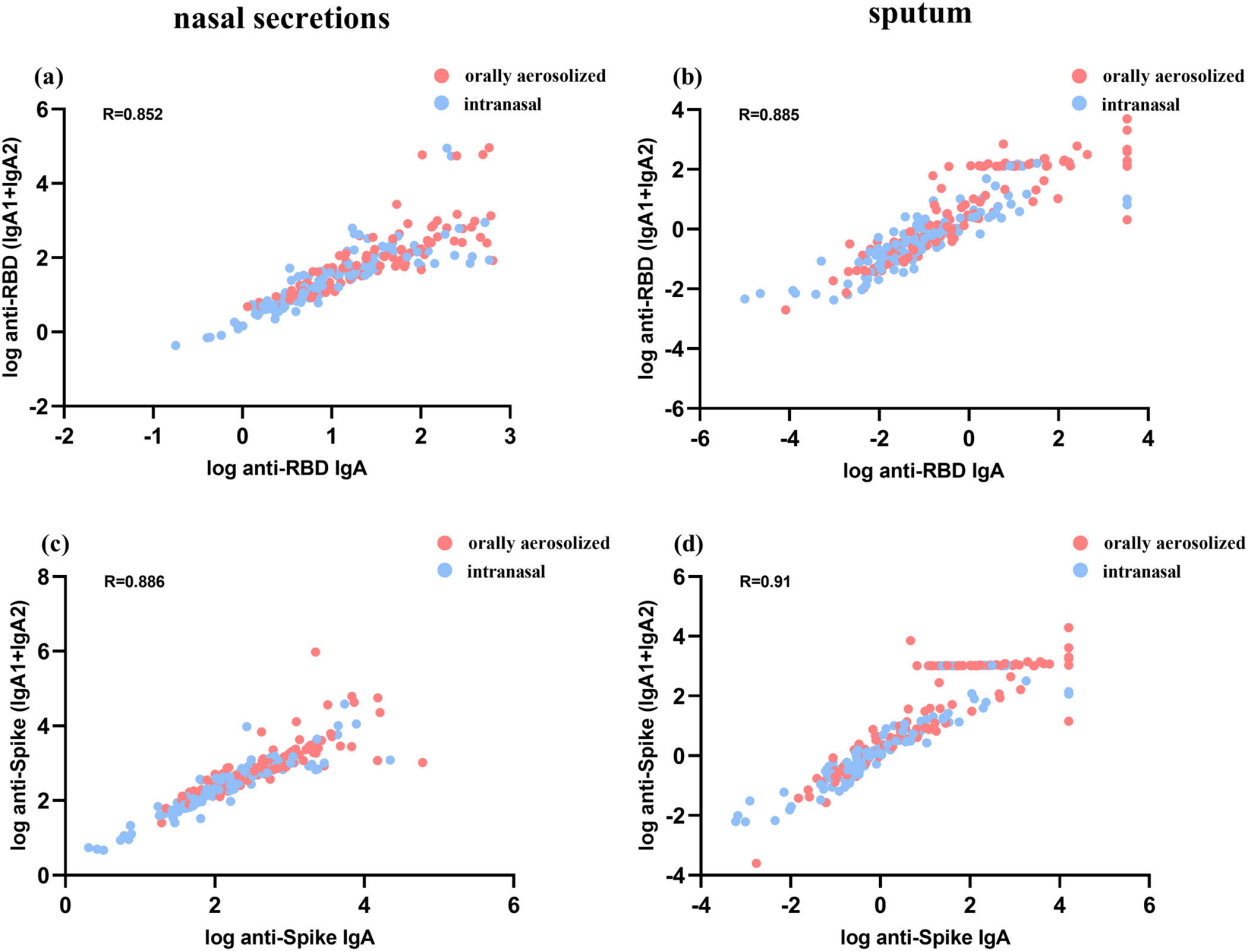
The correlation between the levels of IgA with the combined levels of IgA1 and IgA2 (IgA1 + IgA2). (a and b) represent the correlation between anti‐RBD IgA and IgA1 + IgA2 in nasal secretions and sputum respectively. (c and d) represent the correlation between anti‐Spike IgA and IgA1 + IgA2 in nasal secretions and sputum respectively.

To justify the comparative analysis of IgA1 and IgA2 levels, we evaluated correlations between combined IgA1 + IgA2 levels and total IgA against RBD and Spike antigens in both sample types. Strong linear correlations were observed in nasal secretions (*R* = 0.852, *p* < 0.01) and sputum (*R* = 0.885, *p* < 0.01), confirming that IgA1and IgA2 collectively constitute total IgA (Figure 4). Separate analyses revealed significant correlations between individual subtypes and total IgA, with slightly higher coefficients for IgA1 compared to IgA2 (Table S1).

**Figure 5:**
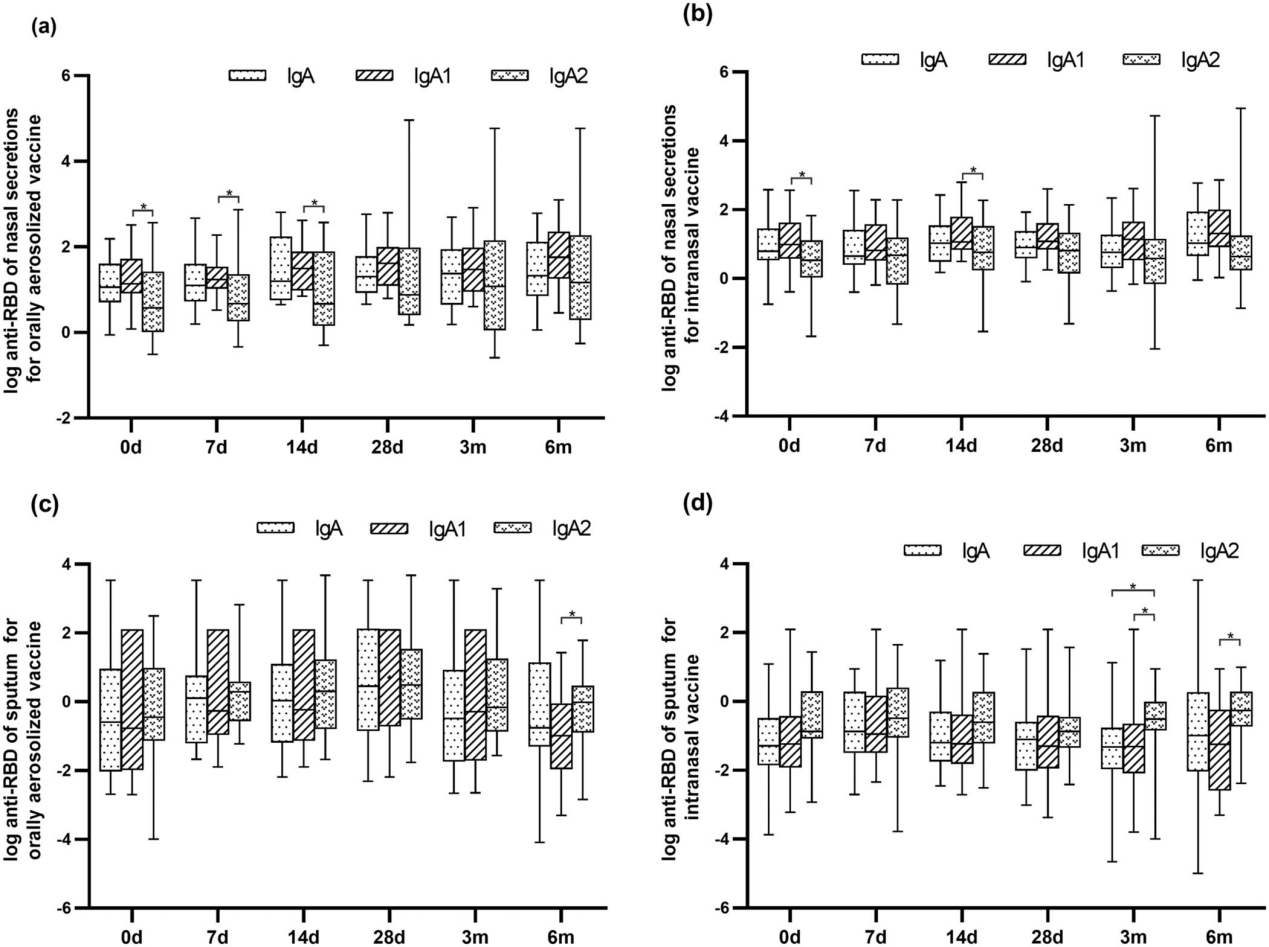
Antibody levels of IgA, IgA1 and IgA2 in nasal secretions and sputum. (a and b) depict the antibody titers of IgA, IgA1, and IgA2 in the nasal secretions and sputum within the orally administered group. (c and d) illustrate the antibody titers of IgA, IgA1, and IgA2 in the nasal secretions and sputum of the intranasal vaccine group. *p*‐values from *t*‐test, comparing each time point shown. *p < 0.05.

Across both vaccination group and most time points, IgA1 levels predominated over IgA2 in both nasal secretions and sputum. Notable exceptions were observed at later time points: Oral aerosolized vaccine: at the 6 months post‐immunization, IgA2 levels exceeded IgA1. Intranasal vaccine: IgA2 levels surpassed IgA1at both 3‐ and 6‐months post‐immunization (Figure 5).

### 3.5 Cytokines Profiling in Nasal Secretions and Sputum

This study aims to investigate the characteristics of cytokine level changes at different time points after vaccination and compare the patterns of mucosal immune cytokine changes induced by different vaccine platforms by analyzing the dynamic changes of cytokines in mucosal secretions.

**Figure 6:**
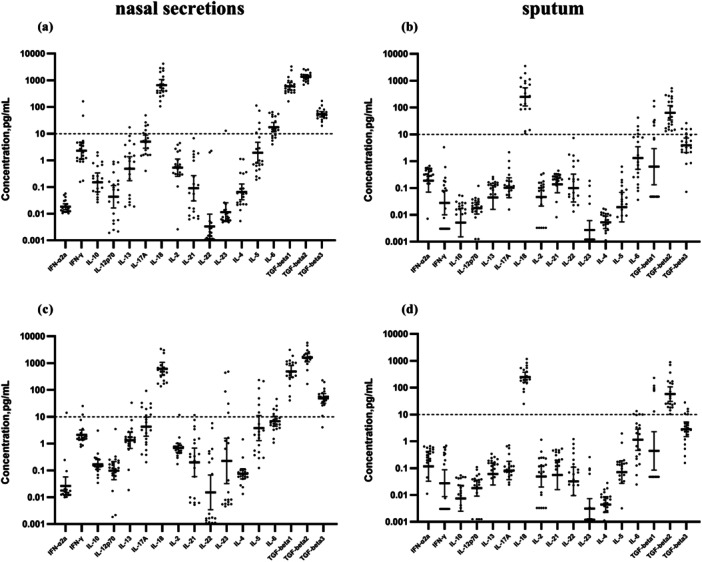
Concentrations of cytokines before immunization. (a and c) represent the baseline levels of cytokines in nasal secretions and sputum of the orally aerosolized vaccine group respectively. (b and d) represent the baseline levels of cytokines in nasal secretions and sputum of the intranasal vaccine group respectively.

All participants were afebrile with no respiratory symptoms at enrollment, despite significantly elevated baseline levels of several cytokines (notably IL‐18 and TGF‐β isoforms) TGF‐β1, TGF‐β2, and TGF‐β3 concentrations were relatively high in nasal secretions, while elevated TGF‐β2 levels were exclusively observed in sputum samples (Figure 6). These findings underscore the importance of normalizing postvaccination cytokine measurements to individual baseline levels.

**Figure 7:**
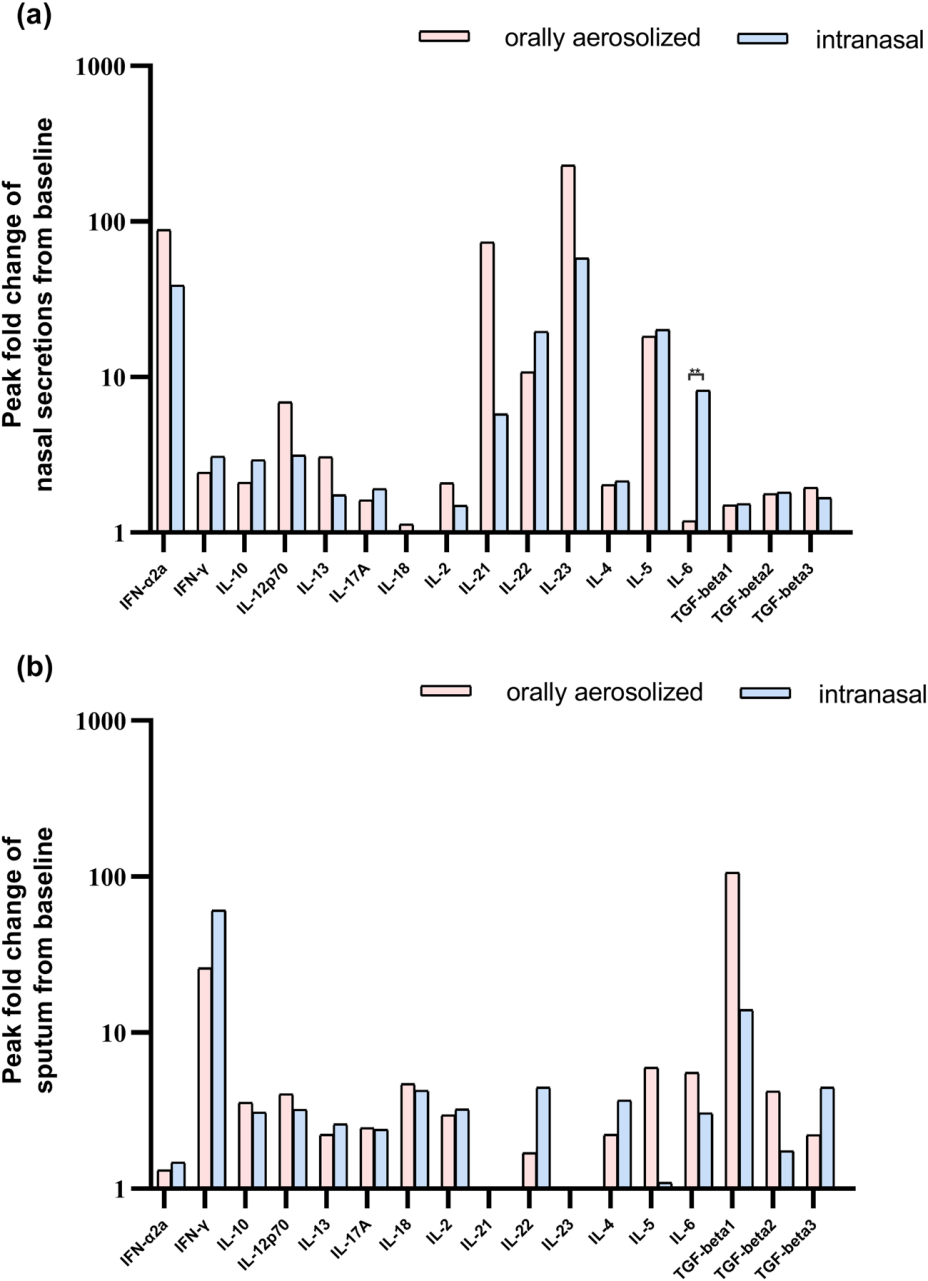
Peak fold change from baseline in levels of cytokines of two groups. The elevation multiples of peak cytokine values at six time points relative to baseline were selected for analysis to compare the levels of two vaccines in nasal secretions (a) and sputum (b), respectively. **p < 0.01.

When comparing cytokine profiles between the two vaccine platforms, a statistically significant difference (*p* < 0.01) was exclusively observed for IL‐6 in nasal secretions. The intranasal vaccine elicited higher IL‐6 levels than the orally aerosolized vaccine. No significant intergroup differences were detected for other cytokines (Figure 7).

**Figure 8:**
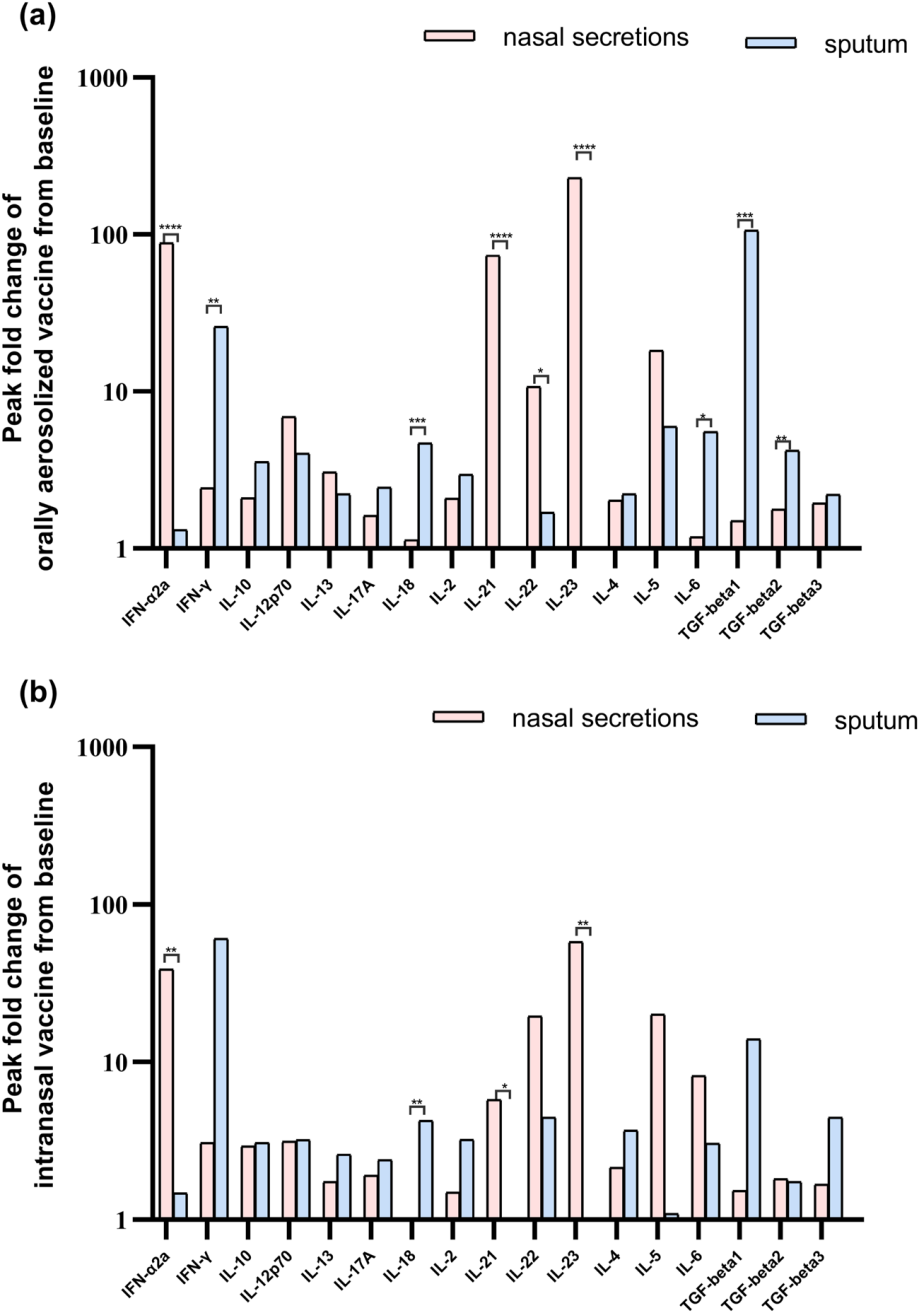
Peak fold change from baseline in levels of cytokines in different samples. The elevation multiples of peak cytokine values at six time points relative to baseline were used to do the analysis. (a) represents the cytokines in the nasal secretions and sputum of the orally aerosolized vaccine group. (b) represents the situation of cytokines in the nasal mucosa and sputum of the intranasal vaccine group. **p < 0.01.

Distinct cytokine distribution patterns between nasal secretions and sputum were identified with each vaccine cohort. Orally aerosolized vaccine group‐Nasal‐predominant cytokines such as IFN‐α2a, IL‐21, IL‐22, and IL‐23 Sputum‐predominant cytokines including IFN‐γ, IL‐18, IL‐6, TGF‐β1, and TGF‐β2 (Figure 8a). For intranasal vaccine group, the nasal‐predominant cytokines include IFN‐α2a, IL‐21, and IL‐23, whereas sputum‐predominant cytokines is IL‐18. No significant differences were observed for other cytokines between compartments (Figure 8b).

Cytokine secretion kinetics were analyzed at 7, 14, 28 days, and 3 months post‐immunization. Fold changes relative to pre‐immunization levels were visualized using heat maps (Figure S1).

In the nasal mucosa both vaccines, IFN‐α2a and IL‐21 exhibited initial increases followed by declines with peaks at Day 28 (orally aerosolized) and Day 14 (intranasal). IFN‐γ showed transient increases (Day 14 for orally aerosolized; Day 7 for intranasal) followed by significant decrease below baseline. TGF‐β1 demonstrated modest declines at Day 7, minor rebounds at Day 14, and subsequent downward trend. IL‐10 levels consistently decreased across all observed time points, whereas IL‐18 and TGF‐β3 showed minimal fluctuations.

Analysis of sputum about oral aerosol vaccine, TGF‐β1 markedly increase at days 7 and 28. IL‐17A increased at Days 14 and 28 but decreased at other time points. IL‐13, IL‐21, and IL‐22 remained consistently below baseline For intranasal vaccine, IL‐13, IL‐17A, and TGF‐β1 increased at Days 7, 14, and 28.

## 4 Discussion

The COVID‐19 pandemic has underscored the critical role of mucosal immunity in preventing respiratory viral infections. This study comparatively analyzed IgA subclass kinetics and cytokine profiles induced by two mucosal vaccines—an orally aerosolized adenovirus vector (Ad5‐nCoV, expressing full‐length spike protein) and an intranasal influenza vector (dNS1‐RBD, expressing RBD domain). Our findings demonstrate that the Ad5‐nCoV induces relatively higher mucosal immune responses compared to the dNS1‐RBD vaccine, as evidenced by higher IgA conversion rates in sputum (65% vs. 40% at day 7 post‐immunization) and sustained IgA2 positivity (50%–65% vs. 35%–50% up to 6 months). Regardless of the vaccine—antibody production in the Lower Respiratory Tract (LRT) is consistently superior to that in the Upper Respiratory Tract (URT).

The observed compartmentalization of mucosal responses may result from Common Mucosal Immune System (CMIS) activation. Orally delivered antigens efficiently prime both nasal‐associated lymphoid tissue (NALT) and bronchus‐associated lymphoid tissue (BALT), promoting synchronized B cell migration to respiratory effector sites. The LRT contains more BALT, supporting greater plasma cell accumulation and IgA secretion [27]. Another reason may relate to the antigen exposure duration. Ad5‐nCoV vaccines maintain longer antigen persistence in the LRT, sustaining prolonged B cell activation compared to intranasal delivery.

An intranasal adenovirus‐vectored vaccine induced a 51.5‐fold increase in nasal secretory IgA (sIgA) after two doses, with sIgA levels inversely correlated with Omicron BA.5 infection risk [28]. This aligns with our observation of 65% IgA conversion in sputum following Ad5‐nCoV vaccination, reinforcing that adenovirus vectors are highly effective for mucosal immunization”.

Although nasal mucosal responses were lower than sputum responses, our sampling methodology significantly improved detection sensitivity. Expandable nasal sponge (5‐min collection) and hypertonic saline‐induced sputum techniques provided higher sensitivity than conventional nasal swabs, which previously yielded only 12%–13% sIgA positivity [7].

These findings gain context when compared to existing mucosal vaccine data—our oral aerosolization route induced 3.2‐fold higher nasal sIgA positivity than intramuscular BBIBP‐CorV vaccination (58% vs. 17%), highlighting the superiority of mucosal delivery platforms [29]. This supports the WHO's strategic priority for developing needle‐free mucosal vaccines to improve global coverage [30].

### 4.1 IgA Subclass Dynamics and Functional Implications

Due to the structural differences in the hinge region and the unique distribution patterns across mucosal surfaces, IgA1 mainly functions in regions such as the respiratory and urogenital tracts. In contrast, IgA2 is predominantly active within the intestinal mucosa. IgA1 binds with the secretory component on the surface of mucosal epithelial cells and is then transported to the mucosal surface as secretory IgA (sIgA), protecting the mucosa by preventing pathogen adhesion to epithelial cells and neutralizing toxins. However, certain bacteria secrete specific IgA1 proteases that cleave IgA1, thus reducing its functional effectiveness. IgA2 is also involved in intestinal mucosal immunity. IgA2 shows structural resistance to enzymatic cleavage by intestinal bacteria, enabling a more stable immune function within the intestinal mucosa [13, 31].

Given these distinct characteristics of IgA1 and IgA2, in this study, the detection of antigen‐specific IgA1 at significantly higher levels than IgA2 in the respiratory tract after vaccination may also be the reason for the suboptimal vaccine efficacy. Although IgA1 has the ability to act as sIgA to play a role in mucosal protection, it is vulnerable to cleavage by certain bacteria‐derived proteases and may have its functionality in the respiratory tract weakened over time. Therefore, its role in providing continuous and powerful defense against pathogens may be compromised. In contrast, the stable nature of IgA2 in the intestinal mucosa is conducive to maintaining the immune response. However, in the respiratory context where IgA1 predominates but is susceptible to enzymatic degradation, the intended immune protection provided by the vaccine may not be fully achieved. This could lead to a situation where the clearance of the pathogen or prevention of reinfection is less effective than expected. In addition, the higher proportion of IgA1 in the respiratory tract may also imply that the immune response pathways that promote IgA2 production are insufficiently activated. Perhaps the vaccine formulation or the mode of antigen presentation fails to effectively stimulate specific immune cells or signaling cascades, resulting in an inability to generate a more balanced or enhanced IgA2 response. Future research efforts could focus on exploring methods to modulate the immune response to increase the relative levels of IgA2 or enhance the stability of IgA1 in the respiratory tract, aiming to optimize the vaccine‐induced immune protection and ultimately improve the overall effectiveness of the vaccine in combating respiratory infections.

### 4.2 Cytokine Networks and Vaccine Optimization

This study compared cytokine profiles in nasal mucosa versus lower‐airway (sputum) samples from the same participants after aerosolized Ad5‐nCoV or intranasal dNS1‐RBD administration, aiming to determine whether the two delivery routes generate distinct cytokine signatures at their target sites (i.e., differences elicited by the two vaccine vectors within the same anatomical compartment; site‐specific differences induced by each vaccine). Mucosal vaccines can induce a mixed cytokine network through NALT and BALT, reflecting the tissue specificity and network coordination of cytokine responses in mucosal vaccination [32, 33, 34]. The cytokine profiles induced by the two vaccines in this study did not show statistically significant differences, and this result needs to be comprehensively interpreted in combination with the complex regulatory network of mucosal immunity and the characteristics of vaccination strategies.

From the research background, multiple studies have confirmed the impact of mucosal vaccination routes on cytokine secretion: oral and intranasal routes can affect cytokine patterns by altering the antigen‐presenting environment, while the polarization differences of innate lymphoid cell (ILC) subsets are the core mechanism by which vaccination routes determine cytokine profiles.

Regarding the results of this study, the lack of significant differences in cytokines may be attributed to three reasons: Firstly, inherent immunostimulatory properties of the viral vectors. Although the two vaccines use different viral vectors (e.g., adenovirus vs. attenuated influenza virus), both are replication‐deficient, which may weaken the cytokine polarization effect mediated by TLR signaling pathways; Secondly, although oral and intranasal routes theoretically trigger different mucosal microenvironments, the high homology of vaccine antigens in this study may narrow the differences in immunogenicity, thereby reducing the differentiation of cytokine secretion.

Despite the absence of significant differences, the potential fluctuations of these cytokines may still affect vaccine efficacy. According to relevant studies, mucosal‐oriented cytokines induced by intranasal vaccination (e.g., IL‐4 that promotes sIgA) may enhance local barrier function, while Th1‐type cytokines associated with oral routes (e.g., IFN‐γ) may be more conducive to maintaining systemic immune memory; meanwhile, the negative feedback regulation between IL‐13 and IFN‐γ suggests that even subtle cytokine imbalances may affect antibody subclass switching (e.g., IgA1/IgA2 ratio) [32, 33]. Future studies can be deepened from three aspects: firstly, expanding the sample size and adopting high‐sensitivity detection technologies (such as single‐cell cytokine sequencing) to capture subtle differences; secondly, combining the NALT immune model to analyze the association between local mucosal cytokines and tissue‐resident immune cells; thirdly, verifying the regulatory effect of vector characteristics on cytokine polarization through in vitro experiments to provide targets for vaccine optimization.

### 4.3 Limitations and Future Directions

This study's limitations include: modest sample size (*n* = 40) with age imbalance (oral cohort: 51.2 ± 8.7 years vs. intranasal: 41.0 ± 6.3 years) and humoral immune responses (including serum IgG and neutralizing antibody titers) were not evaluated, which prevents analysis of the potential interplay between mucosal and systemic immunity. To address this critical issue, complementary experiments are currently underway to: Quantify serum IgG1 and IgG‐3 subclass levels using Determine neutralizing antibody activity against SARS‐CoV‐2 variants.

## 5 Conclusion

The findings of this study indicate that variations in the mucosal immune response at distinct anatomical sites can be attributed to the differing administration routes of respiratory vaccines. This could be connected to the structures and formations of bronchus‐associated lymphoid tissue (BALT) and nasal‐associated lymphoid tissue (NALT). In summary, the immune response elicited by both vaccines was comparatively low, and there were no observed alterations in the secretion levels of IgA‐associated cytokines at any post‐immunization time point. This suggests that conventional vaccine technologies exhibit limitations in effectively targeting mucosal immune‐related cells, such as M cells. Overall, the respiratory mucosal immune system exhibits greater complexity compared to the intestinal immune system. Nonetheless, the research outcomes of the two vaccines have yielded numerous valuable insights, underscoring the considerable progress yet to be made in the advancement of respiratory mucosal immune vaccines.

## Author Contributions

J.W., W.C, Q.Z., and W.Z. conceived the project. J.W., S.B., and J.L. coordinated and performed the cohort study. W.C., W.L., Y.Z, and J.Z. performed the experimental measurements. W.C., A.Z., and J.W. analyzed the data and wrote the manuscript with inputs from all authors.

## Conflicts of Interest

The authors declare no conflicts of interest.

## Supporting information


**Supplement Figure 1:** Heatmap of cytokine changes at different time points for two vaccines. This figure displays the log fold‐change (log (FC)) values of various cytokines in nasal secretions and sputum at different time points (7‐day, 14‐day, 28‐day, and 3‐month) for two groups: orally aerosolized and intranasal. The cytokines measured include TGF ‐ β2, TGF ‐ β1, TGF ‐ β3, IL − 5, IL − 6, IL − 10, IFN ‐ α2a, IFN ‐ γ, IL − 22, IL − 2, IL − 4, IL − 12p70, IL − 13, IL − 21, IL − 23, IL − 17 A, and IL − 18. The log(FC) values are color‐coded for easy interpretation: orange represents log(FC) ≥ 2, gray represents–0.50 < log(FC) < 2, and blue represents log(FC) ≤ − 0.50.

**Supplement Table 1:** The Correlation Coefficient Analysis of IgA1, IgA2 with IgA in Two Vaccine Groups.

## Data Availability

The data that support the findings of this study are available on request from the corresponding author. The data are not publicly available due to privacy or ethical restrictions.
