# Supplementary figures and images for: Kinetics of IgA Subtypes and Cytokines in Respiratory Secretions Following Immunization With COVID‐19 Mucosal Vaccine
Source: J Med Virol. 2025 Oct 13;97(10):e70638. doi: 10.1002/jmv.70638 (PMC12517115; doi:10.1002/jmv.70638)

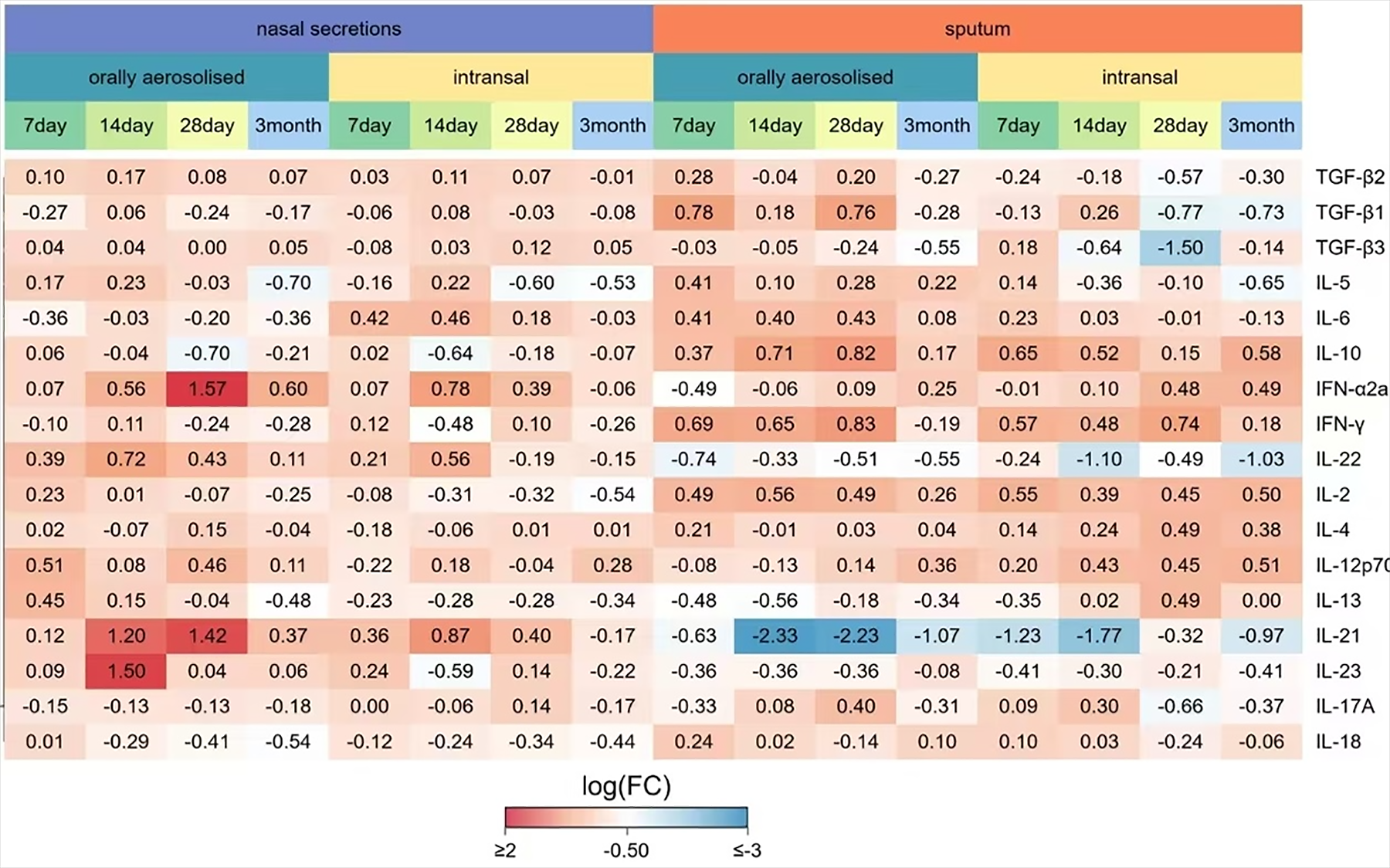

Supplement: Supplementary file 1 — Supplement Figure 1: Heatmap of cytokine changes at different time points for two vaccines. This figure displays the log fold‐change (log (FC)) values of various cytokines in nasal secretions and sputum at different time points (7‐day, 14‐day, 28‐day, and 3‐month) for two groups: orally aerosolized and intranasal. The cytokines measured include TGF ‐ β2, TGF ‐ β1, TGF ‐ β3, IL − 5, IL − 6, IL − 10, IFN ‐ α2a, IFN ‐ γ, IL − 22, IL − 2, IL − 4, IL − 12p70, IL − 13, IL − 21, IL − 23, IL − 17 A, and IL − 18. The log(FC) values are color‐coded for easy interpretation: orange represents log(FC) ≥ 2, gray represents–0.50 < log(FC) < 2, and blue represents log(FC) ≤ − 0.50. [file JMV-97-e70638-s002.tif]
