# Supplementary material for: Kinetics of IgA Subtypes and Cytokines in Respiratory Secretions Following Immunization With COVID‐19 Mucosal Vaccine
Source: J Med Virol. 2025 Oct 13;97(10):e70638. doi: 10.1002/jmv.70638 (PMC12517115; doi:10.1002/jmv.70638)
Supplement: Supplementary file 2 — Supplement Table 1: The Correlation Coefficient Analysis of IgA1, IgA2 with IgA in Two Vaccine Groups. [file JMV-97-e70638-s001.docx]

|  |  | Anti-RBD | | | | Anti-Spike | | | |
| --- | --- | --- | --- | --- | --- | --- | --- | --- | --- |
|  |  | IgA1 | | IgA2 | | IgA1 | | IgA2 | |
|  |  | R | *P* | R | *P* | R | *P* | R | *P* |
| orally aerosolized vaccine | nasal secretions | 0.921 | 0.000 | 0.717 | 0.000 | 0.815 | 0.000 | 0.747 | 0.000 |
|  | sputum | 0.868 | 0.000 | 0.851 | 0.000 | 0.872 | 0.000 | 0.862 | 0.000 |
| intranasal vaccine | nasal secretions | 0.870 | 0.000 | 0.772 | 0.000 | 0.921 | 0.000 | 0.835 | 0.000 |
|  | sputum | 0.626 | 0.000 | 0.545 | 0.000 | 0.903 | 0.000 | 0.835 | 0.000 |

**Supplement Table 1 The Correlation Coefficient Analysis of IgA1, IgA2 with IgA in Two Vaccine Groups**
